# Do early life factors affect the development of knee osteoarthritis in later life: a narrative review

**DOI:** 10.1186/s13075-016-1104-0

**Published:** 2016-09-13

**Authors:** Benny Antony, Graeme Jones, Xingzhong Jin, Changhai Ding

**Affiliations:** 1 Menzies Institute for Medical Research, University of Tasmania, Private Bag 23, Hobart, Tasmania 7000 Australia; 2 Institute of Bone & Joint Translational Research, Southern Medical University, Guangzhou, Guangdong China

**Keywords:** Childhood, BMI, Overweight, Exercise, Fitness, Physical activity, Adulthood, Knee pain, Osteoarthritis

## Abstract

Osteoarthritis (OA) mainly affects older populations; however, it is possible that early life factors contribute to the development of OA in later life. The aim of this review is to describe the association between childhood or early adulthood risk factors and knee pain, structural imaging markers and development of knee OA in later life. A narrative overview of the literature synthesising the findings of literature retrieved from searches of computerised databases and manual searches was conducted. We found that only a few studies have explored the long-term effect of childhood or early adulthood risk factors on the markers of joint health that predispose people to OA or joint symptoms. High body mass index (BMI) and/or overweight status from childhood to adulthood were independently related to knee pain and OA in later life. The findings regarding the association between strenuous physical activity and knee structures in young adults are still conflicting. However, a favourable effect of moderate physical activity and fitness on knee structures is reported. Childhood physical activity and performance measures had independent beneficial effects on knee structures including knee cartilage in children and young adults. Anterior knee pain syndrome in adolescence could lead to the development of patellofemoral knee OA in the late 40s. Furthermore, weak evidence suggests that childhood malalignment, socioeconomic status and physical abuse are associated with OA in later life. The available evidence suggests that early life intervention may prevent OA in later life.

## Background

Osteoarthritis (OA) is characterised by pain, gradual loss of articular cartilage and other structural changes. OA is a disease of the whole joint, eventually leading to total joint replacement. It is the most common joint disorder in adults around the world and it is one of the most frequent causes of pain, loss of function and disability [1]. The global prevalence of radiographically confirmed symptomatic knee and hip OA in 2010 was estimated to be 3.8 % and 0.85 %, respectively [2]. OA is ranked 13th in the top 25 causes of global years lived with disability (DALYs) and the fourth leading cause that showed an increase in DALYs from 1990 to 2013 [3].

Knee OA is the most common form of OA and, currently, there are no registered disease-modifying OA drugs. There is an urgent need for research that investigates innovative and cost-effective approaches to prevent or slow down the progression of knee OA. One of the approaches is to identify and intervene in the early life risk factors that cause this major, but poorly understood, public health problem. The major risk factors associated with this disease are age, female sex, genetic factors, increased body mass index (BMI) and injury. Lifestyle factors such as physical activity and exercise have been associated with knee OA, although the evidence is inconsistent [4–6]. Low socioeconomic position and other co-morbidities, such as cardiovascular diseases, have been considered as risk factors.

OA mainly affects older populations; however, it is possible that early life factors contribute to the development of OA in later life. Considering the mechanical aspect (imbalance in the movement and physical force transmission through the joint) of knee OA, childhood risk factors such as obesity and malalignment could be of great importance in the development of this disease. However, it is difficult to identify the early life risk factors that have a life-course influence on the development of OA due to the difficulties of conducting long-term follow-up from childhood to adulthood. Therefore, only a few studies have reported the childhood risk factors for OA. The aim of this review is to describe the association between childhood or early adulthood risk factors and knee pain, structural imaging markers and the development of knee OA in later life.

## Methods

The literature search involved searching relevant electronic databases (Medline, EMBASE and Google scholar), reference checking, manual searching of relevant journals and recommendations from experts. Due to the low number and diversity of studies and the diversity in outcome measures, a narrative review was felt to be most appropriate to review the relevant literature.

### Early markers of OA

Our understanding of early joint changes associated with OA is increasing due to the use of advanced imaging techniques such as magnetic resonance imaging (MRI). Research on the development and validation of biomarkers, including imaging biomarkers, is a promising method for identifying the early risk factors. Many studies have explored the early life and childhood risk factors in osteoporosis research, which is mainly due to the identification of peak bone mass as a predictor of future osteoporosis or fracture risk [7]. Such markers of joint health are being established in OA research. Cartilage defects, bone marrow lesions (BMLs) and meniscal pathology (early markers of joint abnormalities) are known to occur before the clinical diagnosis of radiographic OA and can predict the development and progression of OA in later life [8]. Tibial cartilage volume in young adults has been proposed as a marker of knee joint health [9, 10] which protects against the development of knee OA in later life (similar to peak bone mass, which is a strong predictor of future risk of osteoporosis in older people) [7]. An ideal approach to identify the early life risk factors in OA is to apply advanced imaging techniques to already existing birth cohorts that have reached adulthood.

Pain is the most common presenting feature of OA, although pain is only weakly related to the structural damage seen in OA [11]. Age, previous knee injuries, overweight and knee-straining work were found to be the risk factors contributing to the incidence of knee pain. These risk factors for self-reported knee pain are similar to the risk factors for knee OA [12]. Therefore, pain should be considered as an important marker, along with other OA features.

## Results

### Early life obesity and adulthood knee OA

Obesity is more associated with knee OA than OA of other joints [13]. Whether childhood obesity leads to later OA has largely been unexplored. The importance of childhood overweight arises from the fact that it predicts adult obesity [14]. Adolescents who were overweight were 18 times more likely than their leaner peers to become obese in early adulthood [15]. Obesity in childhood was strongly predictive of obesity in early adulthood [16]. This tracking of obesity makes it difficult to identify the independent influence of childhood obesity on OA.

Wills and colleagues [17] suggested that obesity from childhood had an accumulative effect on knee OA development. BMI in men as early as 20 years old and women as early as 15 years old was associated with increased risk of knee OA at the age of 53 years [13, 17]. This study suggested that increases in BMI from childhood in women and from adolescence in men was positively associated with knee OA. However, prolonged exposure to high BMI throughout adulthood carried the highest risk and there was no additional risk conferred from adolescence once adult BMI had been accounted for [17]. This study defined knee OA using the symptomatic criteria at 53 years of age and did not have radiographs to confirm the structural pathology. They defined prevalent symptomatic knee OA at one time point (53 years) and could not, therefore, comment on the influence of childhood or adolescent BMI on the incidence of knee OA. The same study group previously reported that hand OA in men at the age of 53 years was associated with increased weight at the age of 26, 43 and 53 years but with decreased weight at birth. The highest risk for hand OA was observed in those who had been heaviest at age 53 years and lightest at birth [18]. However, studies reported that birth weight did not appear to be a major influence on the later development of knee OA in women [19] and the general population [20] after accounting for other confounding factors, including obesity.

BMI in young adults (mean age 23 years) was strongly associated with the incidence of knee OA, but not hip OA, at 65 years of age. For BMI assessed at ages 20 to 29 years, the incidence of knee OA at age 65 years was threefold greater in the heaviest tertile ﻿category than the incidence in the leanest tertile category of BMI after adjustment for age, physical activity and knee injury [21]. BMI at ages 20 to 29 years was more predictive of future OA than at ages 30 to 39 or 40 to 49 years [21].

Using MRI, we reported that overweight children did not differ significantly from normal weight children in knee articular cartilage volume either cross-sectionally or longitudinally over 1.6 years [22]. Similarly, we did not find any significant association between overweight status in childhood and adulthood knee structures, including cartilage volume after 25 years (data not published). However, we found that lean mass in young adults was positively and fat mass was negatively associated with tibial cartilage volume in young Australian adults [23].

#### Early life obesity and adulthood knee joint pain

The knee joint is commonly affected by pain in both overweight paediatric [24] and adult populations [25]. A recent systematic review that studied the relationship between overweight and various musculoskeletal complaints in children suggested that there was moderate evidence for a positive association between overweight during childhood and musculoskeletal pain with a relative risk (RR) of 1.26 (95 % confidence interval (CI) = 1.09–1.97) [26]. However, there was no relationship between body composition or body mass and patellofemoral pain in young school basketball players [27] or knee pain among school children [28].

The effect of childhood obesity on the knee joint can persist into adulthood and lead to higher knee pain in later life. A study by McFarlane et al. on the 1958 British birth cohort explored the association of weight from childhood to adulthood (BMI at 7, 11, 16, 23, 33 and 45 years) with adulthood knee pain at 45 years. They found a higher risk of adulthood knee pain for the obese relative to the underweight subjects in each age group [29]. Childhood BMI was associated with adult knee pain but this association was dependent on adulthood BMI [29]. However, BMI in the early 20s was an independent predictor of knee pain at 45 years [29]. This study had some limitations, for example, adult BMI categories were used for the definition of overweight and obesity in childhood, the reference category for comparison was an underweight group rather than normal weight, men and women were not separated for analyses and they did not use a validated scale for knee pain assessment such as the Western Ontario and McMaster Universities Osteoarthritis Index (WOMAC) scale. A similar study from our group used the Australian Schools Health and Fitness Survey (ASHFS) from 1985 and reported that the childhood overweight measures were significantly associated with adulthood knee mechanical joint pain, stiffness and physical dysfunction among males (aged 31–41 years), independent of the adult overweight measures [30]. Similarly, the change in overweight status from childhood to adulthood was also associated with knee pain; subjects who were overweight in both childhood and adult life had the greatest prevalence and double the risk of knee pain compared with subjects who had normal weight at both time points [30]. Another retrospective life course study on Finnish men (*n* = 1913) who participated in the Health 2000 Study reported that BMI at the age of 20 years increased the likelihood of knee pain and functional limitations of the knee later in life [31]. BMI at the age of 20 years increased the risk of knee pain in either knee by 38 % and functional limitations by 27 % for one standard deviation increment of BMI, respectively. Development of severe obesity in adulthood increased the risk of knee pain by 80 % and functional limitations by 90 %. The effect of obesity on functional limitations was partly mediated by traumatic knee problems during military service [31].

These results support the observation that both systemic and mechanical factors underlie the link between OA and body weight and suggest that developmental influences may be important [18]. For weight-bearing joints, the combination of increased load and changed joint biomechanics is regarded as an underlying mechanism for the association between obesity and OA. Obese individuals show altered biomechanics during everyday movements and these altered biomechanics could initiate OA by changing the load-bearing regions of the articular cartilage in the weight-bearing joints [32]. Systemic factors such as adipokines and other inflammatory markers associated with obesity may also underlie the association of obesity with OA as suggested by a higher prevalence of hand OA (non-weight bearing joint) in obese patients [18, 33].

### Early life joint pain and adulthood knee OA

Knee pain is not an uncommon feature in early life [34]. Studies have found that the prevalence of knee pain is 3.9 % among children and 18.5 % in adolescents [28]. Some studies have reported an even higher prevalence of pathological knee pain in adolescents, with a range from 28.5 to 31 % [35, 36]. Knee pain in early life can result from many conditions that are not related to OA in later life. The most common causes of knee pain in early life include patellar subluxation, Osgood–Schlatter lesion (localised pain at the tibial tuberosity where the quadriceps muscles insert that is mostly associated with a growth spurt), patellar tendonitis, arthritis (bacterial, viral or inflammatory), referred pain from the hip, osteochondritis dissecans, tumours and malignancies. Similarly, anterior knee pain (AKP) is a broad symptom classification which does not imply any particular diagnosis or physical condition and is likely to be multifactorial [37]. Patellofemoral pain syndrome (PFPS) accounts for almost 50 % of nonspecific knee pain in adolescents [38]. Major symptoms of PFPS are diffuse peripatellar and retropatellar localised pain, pain provoked by ascending stairs, descending stairs, squatting, cycling and sitting with flexed knees for prolonged periods of time [39].

Similar to OA in older adults, the prevalence of PFPS has been found to be higher in females, with a prevalence that is approximately 1.5–3 times higher than males in athletic populations [40]. It is possible that these knee pain symptoms persist to later life leading to OA. There is evidence to show that 90 % of AKP sufferers had ongoing problems four years later [41] and 94 % of the AKP cases continued to experience physical difficulties for an average of 8 years following diagnosis [42]. A retrospective study investigating the association between idiopathic AKP in early adulthood and patellofemoral OA found that patients with patellofemoral OA reported more preceding AKP in their adolescence and early adult years than medial unicompartmental tibiofemoral knee OA cases [43]. A systematic review concluded that there was a paucity of high-quality research evidence regarding the link between idiopathic AKP in younger adults and the subsequent development of patellofemoral OA [44]; however, the authors indicated that the evidence from the retrospective case-control study [43] was reliable as its recall bias was minimal and the choice of the control group (medial unicompartmental tibiofemoral knee OA) was suitable. Therefore, AKP can be regarded as one of the early risk factors for knee OA.

### Early life physical activity and adulthood OA

There is consistent evidence that physical activity improves pain and physical function in knee OA and moderate physical activity is recommended by the guidelines for the nonsurgical management of knee OA [45]. However, the effect of physical activity and fitness on the development and progression of OA is controversial as various studies have suggested detrimental [46], beneficial [47] or no effect [48]. The reasons for this controversy are unclear but it may be due to the fact that these studies were retrospective in nature and were not able to take into account the confounding role of injury, which increases the risk of developing OA [49]. It is also possible that knee structures behave differently during different physical activities at different life stages. Weak evidence shows that healthy articular cartilage in vivo responded beneficially to physical loading and degenerated cartilage responded detrimentally [50]. Similarly, in hamsters, physical exercise at a young age had a beneficial effect via enhancing cartilage maturation but had adverse effects on cartilage at a later age with a significant increase in the incidence of OA [51].

Our studies among children aged 9–18 years reported that physical activity was associated with increased cartilage volume both cross-sectionally [52] and longitudinally [22]. In the same study, baseline leg muscle strength was positively associated with cartilage volume accrual and partially mediated the association between physical activity and cartilage volume [22]. Similarly, an interventional study in younger adults (mean age 25 years) using delayed Gadolinium Enhanced MRI of cartilage (dGEMRIC) scans (for detecting the glycosaminoglycan (GAG) content in the cartilage) showed a positive change of dGEMRIC index over 10 weeks in women who were enrolled in a moderate running programme [53]. Similarly, a randomised controlled trial evaluated the effect of moderate exercise on GAG content in knee cartilage and found that the exercise group had an increased GAG content compared with controls in subjects aged between 35 and 50 years [54]. There was a strong correlation (*r* = 0.74) between self-reported physical activity levels and the GAG content in knee cartilage [54]. In contrast, cross-sectional studies of adult triathletes and middle-aged women found no difference in cartilage volume between those who exercised and those who did not, although bone size was generally larger in triathletes than inactive controls [55, 56]. Both studies were small in sample size and cross-sectional in nature. However, in young Australian adults we reported that physical activity measured 5 years before was positively associated with total tibial cartilage volume [57].

A twin study that explored the effect of physical activity on general health found that physical activity reduced the risk of chronic diseases and helped to maintain life satisfaction; however, there was no association between being active or less active and OA in monozygotic or dizygotic twins [58]. A study in preadolescents with lower limb pain reported that 32 % of participants had persistent pain at 1-year follow-up and 31 % reported recurring pain at 4-year follow-up, and vigorous exercise was a significant predictor of lower limb pain persistence at 1-year follow-up but hypermobility was predictive of pain recurrence 4 years later [59]. There was evidence suggesting that athletes had a higher risk of having knee OA features, including osteophytes and radiographic OA, even at a younger age [60].

A recent prospective study from our group in children with a follow-up of 25 years reported that childhood physical performance measures, such as physical work capacity at 170 beats per minute (PWC_170_), were positively associated with adulthood (31–41 years age) tibial cartilage volume independent of adult attained fitness levels [61]. In the same study cohort, we reported that adulthood performance measures, such as leg muscle strength, long jump and PWC_170_ measures, and physical activity measures, such as vigorous, moderate and total activities, were positively associated with adulthood tibial cartilage volume [57]. However, vigorous physical activity in these young adults was weakly but positively associated with BMLs and moderate physical activity was associated with reduced BMLs [62]. Both childhood and adulthood performance measures were associated with tibial bone area and the association between performance measures and cartilage volume was partially mediated by tibial bone area growth, suggesting that greater physical fitness leads to higher cartilage volume through bone development [57, 61].

### Early life injury and adulthood knee OA

Injury to the joint is a major risk factor for OA. Studies have suggested a consistent association between injury and knee OA in all age groups. Only a few studies have explored the association of childhood injury with the incidence of knee OA in later life. The effect of early life injury on OA seems to be smaller than the effect of injury in late adulthood. In a retrospective study, which explored the association between AKP in early adulthood and patellofemoral OA, patients with patellofemoral arthroplasty were found to suffer significantly more patellofemoral instability and trauma in their early adulthood than patients with medial unicompartmental arthroplasty [43]. A study exploring the persistence and recurrence of knee pain in preadolescents found that traumatic lower extremity pain had a 50 % lower risk for pain recurrence compared with non-traumatic pain, indicating a favourable long-term natural course for traumatic pain [59]. Adolescents and young adults (mean age 22) who suffered knee injury were found to be at higher risk of knee OA (RR = 2.95; 95 % CI = 1.35–6.45) at 65 years of age compared with those who did not have injury [49].

There is evidence to suggest that greater BMI in childhood is associated with increased risk of lower extremity injuries and pain [63]. Similarly, increasing BMI is associated with increased risk of lower extremity fractures, such as fractures of the foot, ankle, leg and knee, among children [64]. These injuries and fractures resulting from obesity may lead to knee OA in later life.

### Early life malalignment and adulthood knee OA

Some developmental conditions, such as malalignment, have been proposed as a risk factor for the progression of knee OA, but the prospective evidence is not strong enough to support a causative role of malalignment in the development of knee OA [65]. Self-reported knee malalignment (reported as bow legs) in childhood was associated with cartilage thinning (measured as joint space narrowing using X-ray) in adults over 12 years of follow-up [66]. Similarly, increased risk of isolated knee OA occurred with early adult varus (odds ratio (OR) = 5.16; 95 % CI = 2.87–9.41) and valgus knees (OR = 3.16; 95 % CI = 1.04–9.64) [67]. The authors observed a positive association between knee OA and toe-in foot in this study and this association was explained by varus knee [67]. Although the epidemiological associations are strong, the causal relationship is not established.

There is evidence for a higher prevalence of lower extremity malalignment, fractures and musculoskeletal pain among obese children than children with normal weight [24, 26]. Studies have suggested that the association of obesity with knee OA progression is largely mediated by knee malalignment [68, 69]. Therefore, obesity-related malalignment is important in the context of life-course-approach studies for knee OA. A recent study exploring the knee alignment among obese and non-obese children found that the mean alignment was similar between obese and non-obese subjects [70]; however, in the stratified analysis, there was significantly greater variability in knee alignment among females at higher BMI and greater valgus alignment in obese adolescents in late puberty [70]. A similar study using dual-energy X-ray absorptiometry (DXA) detected a greater prevalence of lower extremity malalignment, mostly valgus deformity, in overweight children [24]. The major limitation of these studies is the use of DXA for assessment of alignment, which needs validation against radiographs. It is possible that mild malalignment with excess weight loaded on the joints over time may contribute to the increased incidence of knee pain and subsequent knee OA. However, further longitudinal studies are required to determine whether childhood obesity is a risk factor for progressive malalignment which predisposes individuals to pain and risk of early OA.

### Early life socioeconomic status and adulthood knee OA

Lower socioeconomic status (SES) has been associated with knee pain, knee OA and total knee replacement in adulthood [71]. Macfarlane and colleagues have identified an association between childhood social status and adulthood wide-spread musculoskeletal pain such as knee pain at 45 years; however, the magnitude of effect of childhood social status on adulthood self-reported pain was less than that of adult social status and was partly explained by poor adult mental health, psychological distress, adverse life events and lifestyle factors [72]. Similarly, there was an independent association of both childhood and current SES with self-reported arthritis [73]. Increased BMI was the most likely mechanism underlying the association between childhood SES and arthritis onset [73].

### Early life abuse and adulthood OA

Childhood abuse could be physical, sexual, emotional and/or neglect. Previous research has identified an association between childhood abuse and arthritis in adulthood [74, 75] and this association was strongest and most consistent with childhood physical abuse. The evidence of a relationship between childhood sexual abuse and arthritis is conflicting; one study showed an association [76] and another did not [75]. Individuals who reported a diagnosis of OA had significantly higher odds (OR = 1.99; 95 % CI = 1.57–2.52) of reporting childhood physical abuse [77]. This association remained significant even after controlling for demographic and SES characteristics, in addition to childhood stressors, adult health behaviours and mood disorders, which were associated with childhood physical abuse and arthritis.

## Conclusions

Although the evidence for associations between childhood or early life factors and OA is limited, the existing evidence suggests that obesity from early life is independently associated with knee OA and knee pain. The evidence for injury, malalignment, childhood SES and childhood abuse are mostly from retrospective studies and such associations need to be confirmed by prospective studies. Anterior knee pain syndrome in adolescence could lead to the development of patellofemoral knee OA in the late 40s. The findings regarding the association between strenuous physical activity and knee structures in young adults are still conflicting. However, a favourable effect of moderate physical activity and fitness on knee structures is reported. Childhood physical activity and fitness may have an independent beneficial effect on knee joint health. These data suggest that early life intervention may prevent OA in later life. However, further cohort studies with larger sample sizes and long-term follow-ups are required to determine if OA in later life stems from childhood or early adulthood risk factors.

## Abbreviations

AKP: Anterior knee pain
BMI: Body mass index
BML: Bone marrow lesion
CI: Confidence interval
DALYs: Disability adjusted life years
dGEMRIC: Delayed gadolinium enhanced MRI of cartilage
DXA: Dual-energy X-ray absorptiometry
GAG: Glycosaminoglycan
MRI: Magnetic resonance imaging
OA: Osteoarthritis
OR: Odds ratio
PFPS: Patellofemoral pain syndrome
PWC_170_: Physical work capacity at 170 beats per minute
RR: Relative risk
SES: Socioeconomic status
